# Re-evaluation of MEFV carriers previously diagnosed with FMF: a colchicine discontinuation study

**DOI:** 10.1093/rheumatology/keaf340

**Published:** 2025-06-13

**Authors:** Veysel Cam, Dilara Unal, Erdal Sag, Yagmur Bayindir, Emil Aliyev, Hulya Ercan Emreol, Mehmet Orhan Erkan, Ozlem Necipoglu Banak, Hazel Delal Dara Kar, Ozge Basaran, Yelda Bilginer, Seza Ozen

**Affiliations:** Department of Paediatrics Rheumatology, Hacettepe University Faculty of Medicine, Ankara, Turkey; Department of Paediatrics Rheumatology, Hacettepe University Faculty of Medicine, Ankara, Turkey; Department of Paediatrics Rheumatology, Hacettepe University Faculty of Medicine, Ankara, Turkey; Department of Paediatrics Rheumatology, Hacettepe University Faculty of Medicine, Ankara, Turkey; Department of Paediatrics Rheumatology, Hacettepe University Faculty of Medicine, Ankara, Turkey; Department of Paediatrics Rheumatology, Hacettepe University Faculty of Medicine, Ankara, Turkey; Department of Paediatrics Rheumatology, Hacettepe University Faculty of Medicine, Ankara, Turkey; Department of Paediatrics Rheumatology, Hacettepe University Faculty of Medicine, Ankara, Turkey; Department of Paediatrics Rheumatology, Hacettepe University Faculty of Medicine, Ankara, Turkey; Department of Paediatrics Rheumatology, Hacettepe University Faculty of Medicine, Ankara, Turkey; Department of Paediatrics Rheumatology, Hacettepe University Faculty of Medicine, Ankara, Turkey; Department of Paediatrics Rheumatology, Hacettepe University Faculty of Medicine, Ankara, Turkey

**Keywords:** FMF, *MEFV* mutation, heterozygous carriers, colchicine discontinuation

## Abstract

**Objectives:**

FMF is an autoinflammatory disease associated with mutations in the MEFV gene. While typically inherited in an autosomal recessive pattern, heterozygous individuals may also exhibit FMF symptoms, often with a milder disease course. The long-term management of colchicine therapy in heterozygous patients, particularly decisions regarding its discontinuation, remains a clinical challenge.

**Methods:**

This retrospective cohort study evaluated paediatric patients with a heterozygous pathogenic MEFV mutation who were followed at a single tertiary centre between September 2024 and March 2025. Both patients in whom colchicine therapy was successfully discontinued and those in whom discontinuation was not feasible were analysed. Clinical characteristics, attack features, inflammatory markers and treatment outcomes were assessed. Multivariate logistic regression and ROC curve analyses were performed to identify predictors of successful colchicine discontinuation.

**Results:**

A total of 136 patients were included. Of the 84 patients who attempted colchicine discontinuation, 72 (85.7%) remained off therapy, while 12 (14.3%) resumed treatment. Early absence of attacks during follow-up was associated with successful colchicine discontinuation, whereas arthritis predicted continued treatment. ROC analysis showed that a ≥70.8% reduction in attack frequency during the first six months of therapy strongly predicted successful discontinuation (AUC = 0.883, 95% CI: 0.823–0.943).

**Conclusion:**

Our findings suggest that colchicine therapy can be safely discontinued in selected heterozygous individuals who show early absence of attacks, suggesting that the initial diagnosis of FMF in some patients may warrant reconsideration. However, it is important to closely monitor these children after treatment cessation, and decisions should be guided by careful follow-up and regular reassessment.

## Graphical abstract

**Figure:**
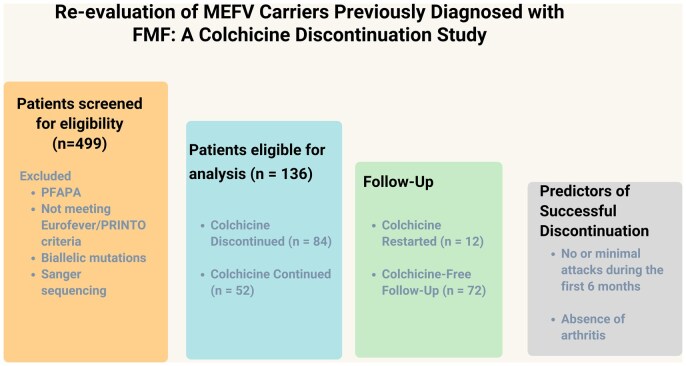

Rheumatology key messages

- Heterozygous MEFV carriers diagnosed with FMF should be periodically re-evaluated after colchicine initiation to confirm the accuracy of the diagnosis.
- Sustained inactive disease over time warrants reassessment of both the initial diagnosis and the need for ongoing colchicine therapy.

## Introduction

FMF is an autoinflammatory disease predominantly seen in populations of Mediterranean origin [1]. Clinically, it is characterized by recurrent episodes of fever, peritonitis, pleuritis and arthritis. FMF is associated with mutations in the *MEFV* gene and is classically inherited in an autosomal recessive pattern. However, several studies have reported that individuals carrying a single pathogenic *MEFV* mutation (i.e. heterozygotes) may also present with clinical manifestations of FMF [2]. In such cases, the disease tends to follow a milder course, and the risk of developing amyloidosis appears to be lower [3].

In light of these observations, experts from ISSAID/EMQN have proposed a distinction between patients with a confirmative and a non-confirmative genotype for FMF. Individuals who are homozygous or compound heterozygous for *MEFV* mutations are considered to have a confirmative genotype, whereas those carrying a single pathogenic mutation or biallelic variants of unknown significance (VUS) are classified as having a non-confirmative genotype [4].

Building on this distinction, the Eurofever/PRINTO classification criteria require that one clinical feature is sufficient to classify individuals with a confirmative genotype as FMF, whereas patients with a non-confirmative genotype must present with at least two clinical features consistent with the disease [5].

The long-term management of heterozygous patients, including decisions regarding the continuation or discontinuation of colchicine therapy, remains a clinical challenge. Some studies have reported successful colchicine withdrawal in selected heterozygous patients who maintained prolonged remission. However, symptom recurrence following treatment cessation has also been described.

At our centre, the necessity of ongoing colchicine therapy in heterozygous FMF patients who achieve long-term remission has become a significant clinical question. Accordingly, we consider discontinuation of colchicine in patients who have remained attack-free for at least 2 years. Depending on follow-up conditions, this duration may be extended to up to 4 years, and in rare instances, even longer. Our objective was to select a cohort of heterozygous patients in whom colchicine could be discontinued to assess predictive factors associated with successful treatment cessation and, consequently, potential misdiagnosis.

## Methods

This retrospective, observational cohort study was conducted at a single tertiary paediatric rheumatology centre. We screened paediatric patients carrying a single pathogenic heterozygous mutation in the MEFV gene, who were regularly followed in our clinic, for eligibility.

**Figure 1.**
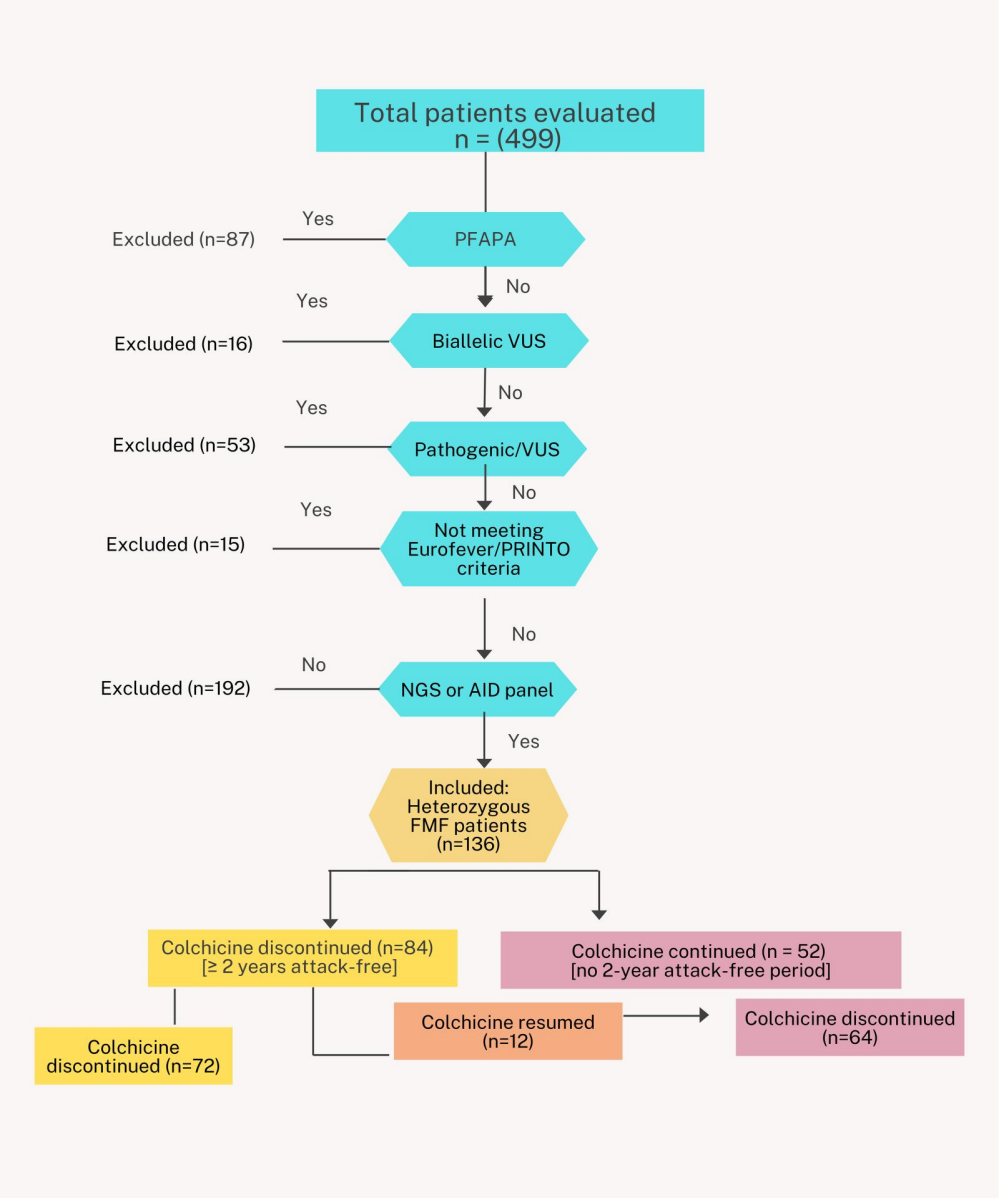
Flowchart of patient selection and inclusion criteria. Out of 499 patients, 363 were excluded due to PFAPA diagnosis, biallelic VUS, pathogenic/VUS combinations, not meeting Eurofever/PRINTO criteria or lack of genetic panel testing. The remaining 136 heterozygous FMF patients included 84 who discontinued colchicine after at least two years without attacks; 12 of them later resumed treatment. In 52 patients, colchicine was continued due to the absence of a 2-year attack-free period

Patients were eligible for inclusion if they: (i) had undergone full sequencing of the MEFV gene, (ii) carried only one pathogenic mutation, (iii) had no clinical attacks during at least 2 years prior to colchicine discontinuation and (iv) had regular monitoring of inflammatory markers (SAA, CRP, ESR). Patients were excluded if full gene sequencing was not performed, if they met the clinical profile of PFAPA syndrome, or if they did not meet Eurofever/PRINTO FMF classification criteria (Fig. 1).

To ensure that patients had both a long follow-up period and a recent clinical evaluation, we included those who were first registered in our clinic on or after December 2015 and had an outpatient follow-up visit between September 2024 and March 2025. This 6-month window ensured that only patients who remained under active clinical care were evaluated. The screening and data collection were completed in April 2025.

Demographic and clinical data—including age at symptom onset, family history, consanguinity and attack characteristics—were extracted from electronic medical records. Attack frequency and inflammatory markers were recorded at three defined time points: (i) prior to colchicine discontinuation, (ii) at the first follow-up after discontinuation and (iii) at the final clinical visit. Patient-reported attack diaries were also reviewed.

Both patients who successfully discontinued colchicine and those in whom discontinuation failed were included. Adherence to treatment was not systematically evaluated; however, all included patients had regular follow-up and were assessed for inflammatory activity at each visit.

Patients with missing inflammatory marker data at any of the three time points were excluded. Missing demographic data were not imputed and were treated as missing during analysis.

### Statistical analysis

All analyses were conducted using IBM SPSS Statistics for Windows, Version 26.0 (IBM Corp., Armonk, NY). Continuous variables were presented as mean ± standard deviation (SD) or median with interquartile range (IQR), depending on the distribution assessed by the Shapiro–Wilk test. Categorical variables were reported as frequencies and percentages (*n*, %).

Group comparisons for categorical variables (e.g. clinical features between colchicine-discontinued and colchicine-continued groups) were performed using Pearson’s *χ*^2^ test or Fisher’s exact test, depending on expected cell counts. For continuous variables, the independent samples *t*-test was used for normally distributed data, and the Mann–Whitney *U* test was used for non-normally distributed data.

To identify variables that could predict colchicine discontinuation, multivariate binary logistic regression analysis was performed. Variables with a *P*-value <0.10 in univariate analyses were included in the multivariate model using the enter method.

Additionally, receiver operating characteristic (ROC) curve analysis was used to assess whether the reduction in attack frequency during the first 6 months of treatment could predict colchicine discontinuation. The area under the curve (AUC) and 95% CIs were calculated, and the optimal cut-off value was determined using the Youden index.

A two-sided *P*-value <0.05 was considered statistically significant for all tests.

The study has received approval from the Ethics Committee (SBA 25/251, dated March 24, 2025). Due to the retrospective nature of the study and the anonymization of patient data, the ethics committee waived the requirement for informed consent.

## Results

A total of 136 patients were included in the analysis, however 52 did not meet the criteria defined above and thus colchicine was not stopped. Of the remaining 84 patients who attempted colchicine discontinuation, 12 later resumed treatment (14.2%).

Among patients who successfully discontinued colchicine, 27.8% were female, with a median age at symptom onset of 5 years (IQR 4–7 years). The most common MEFV mutation was M694V/WT, detected in 70.9% of patients, followed by M680I/WT (16.7%) and V726A/WT (8.3%). Fever was the most frequent attack feature observed in all patients (100%), followed by abdominal pain (87.5%) and myalgia (36.1%). Chest pain (4.1%) and arthritis (9.7%) were less common. Exercise (27.7%) was the most frequent trigger reported for attacks.

The baseline characteristics of the entire cohort are summarized in Table 1.

**Table 1.** Summary of demographic and clinical variables at baseline

Characteristics	Discontinued colchicine	Continued colchicine	All patients
Female, *n* (%)	20 (27.8)	46 (71.9)	66 (48.6)
Age at symptom onset, years, median (25th–75th)	5 (4–7)	5 (4–6)	5 (4–7)
MEFV mutations, *n* (%)			
Heterozygous			
M694V/WT	51 (70.9)	48 (75)	99 (72.8)
M680I/WT	12 (16.7)	6(9.4)	18 (13.2)
V726A/WT	6 (8.3)	10 (15.6)	16 (11.8)
F479L/WT	3 (4.1)	0	3 (2.2)
Attack duration, days, median (25th–75th)	3 (2–3)	3 (2–3)	3 (2–3)
Positive family history for FMF, *n* (%)	30 (41.6)	28 (43.7)	58 (42.6)
Parental consanguinity, *n* (%)	5 (7.5)	7 (13.2)	12 (10)
Attack characteristics, *n* (%)			
Fever	72 (100)	58 (90.6)	130 (95.6)
Abdominal pain	63 (87.5)	50 (78.1)	113 (83.0)
Chest pain	3 (4.1)	19 (29.6)	22 (16.1)
Arthritis	7 (9.7)	21 (32.8)	28 (20.5)
Myalgia	26 (36.1)	37 (57.8)	63 (46.3)
Erysipelas-like erythema	2 (2.7)	2 (3.1)	4 (2.9)
Triggers, *n* (%)			
Exercise	13 (21.3)	20 (40.8)	33 (27.5)
Seasonal change	1 (1.6)	3 (6.1)	4 (3.0.3)
Stress	7 (11.5)	10 (20.4)	17 (14.2)
Menstruation	0 (0.0)	2 (4.1)	2 (1.7)
Attacks prior to treatment (6 months), median (25th–75th)	5 (4–6)	6 (3–6)	6 (4–6)
Attacks after treatment (6 months), median (25th–75th)	0 (0–1)	2(1–2)	1 (0–2)

The mean age at which colchicine was discontinued was 10.06 ± 2.65 years. The attack-free duration before colchicine stop was as follows: Of the patients, 32.2% had 2 years, 26.2% had 3 years, 28.5% had 4 years, 10.7% had 5 years and 2.4% had 6 years or more of follow-up. The 12 patients who resumed colchicine treatment had discontinued it after a 2–4-year attack-free period. The mean follow-up duration after treatment discontinuation in patients who did not resume colchicine was 3.46 ± 1.98 years. The mean duration before colchicine was resumed was 1.9 ± 1.1 years.

### Univariate analysis

In the univariate analysis, the relationship between colchicine discontinuation and factors such as fever, myalgia, abdominal pain, chest pain, symptom onset age and treatment response were analysed. Fever was strongly associated with colchicine discontinuation (*χ*^2^(1, *N* = 136) = 7.062, *P* = 0.008; Fisher’s exact *P* = 0.010). However, fever was present in nearly all patients in both the groups, and the effect size was limited (100% in the colchicine-discontinued group and 90.6% in the continued group, 0.23). Chest pain was significantly associated with colchicine discontinuation (*χ*^2^(1, *N* = 136) = 15.998, *P* < 0.001). Arthritis was similarly significantly related to colchicine discontinuation (*χ*^2^(1, *N* = 136) = 11.049, *P* = 0.001). Myalgia showed a significant relationship (*χ*^2^(1, *N* = 136) = 6.074, *P* = 0.014), though its effect size was small. Attack reduction percentage was also significantly associated with colchicine discontinuation (*U* = 539.500, *P* < 0.001). Symptom age was significantly associated with colchicine discontinuation (*P* = 0.012), with younger patients more likely to discontinue colchicine. The median attack frequency during the 6 months preceding colchicine initiation was comparable between the two groups (*P* = 0.774). Likewise, no statistically significant difference was observed in median attack duration (*P* = 0.772). The results of the univariate analysis are summarized in Table 2.

**Table 2.** Univariate and multivariate analysis of factors associated with colchicine discontinuation

Variable	Test statistic (univariate)	*P*-value (univariate)	*B* (multivariate)	*P*-value (multivariate)	Exp(*B*)
Pre-treatment attack frequency	2367.500 (*U*)	0.774	–	–	–
Attack duration	2244.000 (U)	0.772	–	–	–
Arthritis	11.049 (*χ*²)	0.001*	−1.853	0.013*	0.157
Myalgia	6.074 (*χ*²)	0.014*	−1.019	0.055	0.361
Attack reduction percentage	539.500 (*U*)	<0.001*	−12.149	<0.001*	≈0.000
Symptom age	2211 (*U*)	0.682	−0.213	0.040*	0.808
Chest pain	16.000 (*χ*²)	<0.001*	−1.630	0.049*	0.196

*U*: Mann–Whitney *U* test statistic; *χ*^2^: *χ*^2^ test statistic; *B*: regression coefficient; Exp(*B*): exponentiated *B* coefficient (odds ratio)

* *P*-values <0.05 are considered statistically significant.

### Multivariate analysis

In the multivariate logistic regression analysis, variables that were statistically significant in the univariate analysis were included: fever, chest pain, arthritis, myalgia, symptom onset age and percentage reduction in attack frequency. Fever was excluded from the final model due to quasi-complete separation, as all patients who discontinued colchicine had a history of fever, leading to instability in coefficient estimates (*B* = 18.574, SE = 15 675.385, *P* = 0.999) (Supplementary Table S1, available at *Rheumatology* online).

Reduction in attack frequency observed during early follow-up was the strongest predictor of successful colchicine discontinuation (*B* = −12.149, SE = 2.173, *Z* = −7.962, *P* < 0.001, Exp(*B*) ≈ 0.000). Symptom onset age was also a significant predictor (*B* = −0.213, SE = 0.104, *Z* = −4.199, *P* = 0.040, Exp(*B*) = 0.808). The presence of arthritis significantly reduced the likelihood of colchicine discontinuation, while chest pain showed a weaker but still negative association. Myalgia (*B* = −1.019, SE = 0.530, *Z* = −1.960, *P* = 0.055, Exp(*B*) = 0.361) showed borderline significance (Table 2).

**Figure 2.**
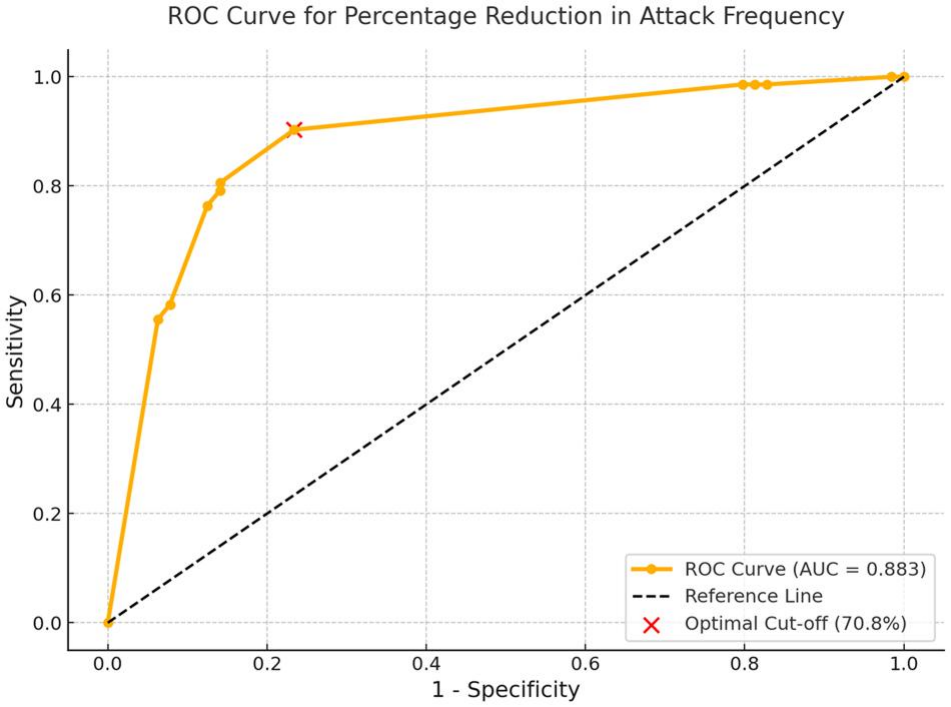
ROC curve for percentage reduction in attack frequency. The ROC analysis shows that percentage reduction in attack frequency predicts colchicine discontinuation with an accuracy of 88.3% (AUC = 0.883). The optimal cut-off was identified as 70.8%, where sensitivity and specificity are best balanced

Based on the results of the multivariate logistic regression model, ROC analysis was conducted for the variable ‘percentage reduction in attack frequency during the first 6 months of treatment’, which showed a strong predictive value for successful colchicine discontinuation. The analysis revealed high discriminative performance (AUC = 0.883, 95% CI: 0.823–0.943, *P* < 0.001). According to the Youden index, the optimal cut-off value was 70.8%, with a sensitivity of 90.3% and specificity of 76.6%, suggesting that patients achieving this threshold may have an increased likelihood of treatment withdrawal (Fig. 2).

## Discussion

FMF is an autosomal recessive disease. However, a certain percentage of patients diagnosed with FMF carry only one mutation. Thus, the presence of a heterozygous mutation poses a challenge for clinicians when these patients present with fever and/or inflammatory manifestations. In children, fever and abdominal pain are very frequent features; functional abdominal pain is particularly common. Moreover, abdominal pain may accompany many common infections in childhood. On the other hand, Lachmann *et al.* have shown that baseline CRP levels in carriers of MEFV mutations are higher than those in the healthy population, and we have shown that the fever response in carriers is more intense [6, 7]. Therefore, a Turkish child who experiences fever attacks and abdominal pain, and carries one MEFV mutation, can easily be misdiagnosed with FMF. Our aim was to identify heterozygous patients in whom treatment could be successfully stopped. These findings suggest that some patients may have been misclassified at the time of diagnosis. In addition, the absence of reported attacks in long-term follow-up may also reflect improved parental understanding of disease manifestations, leading to more accurate differentiation between true FMF attacks and other non-specific symptoms over time. Furthermore, the absence of arthritis was identified as a predictive factor for successful colchicine discontinuation.

In homozygous patients, colchicine treatment is typically maintained for life. This necessitates daily, long-term medication, which can be associated with the psychological burden of managing a chronic illness. However, in heterozygous patients, the duration of colchicine treatment and which patients should continue treatment for longer periods or for life remain unclear. In the 2016 EULAR recommendations for FMF management, it was suggested that stopping treatment could be considered in heterozygous cases after 5 years of being attack-free [8]. The new recommendations 2024 EULAR updated proposals indicate that treatment should not be discontinued in patients with two mutations [9]. There is a considerable amount of literature on heterozygous FMF cases, with many studies investigating the course of the disease and treatment approaches. An earlier study by Hentgen *et al.* demonstrated that some heterozygous patients entered remission in adulthood [10]. A study by Sonmez *et al.* from our group, involving a cohort of 112 patients, including 22 who discontinued colchicine, reported that two of these patients later resumed treatment [11]. The average attack-free period before discontinuation was 27 months in this cohort. Furthermore, the study found that the colchicine-discontinued group had higher attack frequency and annual attack rates. In a more recent study by Cohen *et al.*, colchicine was discontinued in 51 patients, but 30 of them later resumed treatment [12]. The study linked the need for treatment resumption to severe disease and shorter remission periods. However, unlike our study, this research also included compound heterozygous patients.

Elhani *et al.* investigated 52 patients who discontinued colchicine, with 23 of them later resuming treatment [13]. Similarly, Butbul *et al.* examined 59 patients who discontinued colchicine [14]. This study had a long follow-up period (5.0 ± 3.05 years), during which 11 patients (20%) experienced a relapse. In these patients, treatment was resumed after a median of 0.7 years (range: 0.3–5.0 years). Arthritis and myalgia were more common in this group. A longer attack-free period before colchicine discontinuation was found to predict successful treatment cessation. Furthermore, a study by Tanatar *et al.*, which represents one of the largest series to date, included 64 patients who discontinued colchicine, with 17 eventually restarting treatment. Notably, patients who resumed treatment were younger at the time of colchicine discontinuation and had shorter attack-free durations prior to cessation [15].

The studies mentioned suggest that colchicine treatment may be discontinued in at least some patients, with a longer attack-free period serving as a predictive factor for discontinuation. Additionally, these studies indicate that the presence of conditions such as arthritis, myalgia or severe disease classification are associated with continued treatment. These findings underline the importance of identifying specific patient characteristics that may influence the decision to either discontinue or continue colchicine therapy.

In our study, the median attack duration was not a significant factor. While attack duration was highlighted as a key factor in previous studies, it did not emerge as a major determinant in our study. Similarly, the median number of attacks in the 6 months prior to treatment initiation did not significantly differ between the groups. The median symptom age was slightly higher in the colchicine-discontinued group; however, both the groups fell within the preadolescent age range, and the medians were closely aligned, suggesting limited clinical relevance. In scoring systems developed to assess disease severity, arthritis, which suggests more severe disease, was associated with continued treatment [16]. Chest pain, a milder indicator, was also a contributing factor. However, the most pronounced predictor of colchicine discontinuation was a marked decrease in attack frequency during follow-up, particularly within the first 6 months. A significant proportion of patients who entered remission experienced a ≥75% reduction in attack frequency during the first 6 months of follow-up. Although the recommendation is to discontinue colchicine after 5 years of being attack-free, colchicine was often discontinued after shorter periods in other studies. Similarly, in our study, a significant proportion of patients discontinued treatment after 2–4 years of being attack-free, with the timing of treatment resumption being homogeneously distributed. As a final point regarding our findings, it should be noted that the discontinuation rates observed in this study may not fully reflect the entire cohort, as patients in whom only a limited number of mutations were tested by Sanger sequencing were excluded.

While these encouraging results suggest the potential for successful colchicine discontinuation in certain cases, it should be noted that adult studies have demonstrated the development of amyloidosis even in heterozygous patients. However, in those studies, full sequencing of the *MEFV* gene was not performed, and only known pathogenic mutations were evaluated, raising the possibility that undetected additional mutations may have contributed to disease progression [17]. On the other hand, in a series of secondary amyloidosis cases from France, 15.3% of the patients did not have any mutations in the *MEFV* gene or in other autoinflammatory disease (AID) genes, suggesting that unknown factors may contribute to the development of amyloidosis.

We do not attempt discontinuation in patients who are unlikely to adhere to clinical follow-up with monitoring of acute-phase reactants. Therefore, even if attack-free remission is achieved, if there are concerns regarding follow-up compliance, we agree that colchicine discontinuation should be approached with caution.

In such cases, it is essential to emphasize the importance of regularly monitoring markers such as SAA and CRP to track both disease progression and subclinical inflammation.

Although we focused on colchicine discontinuation, early FMF diagnoses based on non-specific symptoms such as fever and abdominal pain—which may overlap with functional abdominal pain or infections—might have led to misclassification in some patients.

As some patients were referred from other centres, a multidisciplinary team decision may be helpful when considering colchicine treatment in heterozygous patients.

This should be taken into account when interpreting our results.

This study has certain limitations. First, its retrospective nature may introduce constraints regarding data completeness and follow-up consistency. Second, the median follow-up period after colchicine discontinuation was 3.26 years, which is shorter than the 5-year duration suggested in previous studies. Additionally, as this was a single-centre study, further validation through multicentre prospective studies is needed to support the generalizability of these findings.

These results should be confirmed by prospective studies including at least 5 years of follow-up after colchicine discontinuation.

## Conclusion

In this study, consistent with the existing literature, we demonstrated that some heterozygous patients can achieve long-term remission and can be safely monitored without colchicine therapy. Arthritis was associated with the continuation of colchicine treatment, whereas the lack of attacks predicted successful treatment discontinuation. Thus, it should be considered that some individuals with one mutation who have some inflammatory manifestation at one point in their childhood may not necessarily have FMF. Therefore, we emphasize that colchicine therapy, once initiated, does not necessarily need to be continued for life in all cases. We need biomarkers or omics signatures that would help us define the heterozygotes who truly express the FMF phenotype and require colchicine.

## Supplementary Material

keaf340_Supplementary_Data

## Data Availability

The datasets generated and analysed during the current study are available from the corresponding author on reasonable request.
