# Supplementary material for: Re-evaluation of MEFV carriers previously diagnosed with FMF: a colchicine discontinuation study
Source: Rheumatology (Oxford). 2025 Jun 13;64(11):5726–32. doi: 10.1093/rheumatology/keaf340 (PMC12596059; doi:10.1093/rheumatology/keaf340)
Supplement: keaf340_Supplementary_Data [file keaf340_supplementary_data.docx]

Supplementary Material

# Supplementary Table S1. Full Logistic Regression Model (Raw Output)

Note: Fever yielded extreme coefficient values and standard errors, indicating model instability likely due to sparse data or quasi-complete separation.

| Variable | B | S.E. | Wald | df | Sig. | Exp(B) |
| --- | --- | --- | --- | --- | --- | --- |
| Attack Reduction (%) | -11.803 | 2.191 | 29.017 | 1 | .000 | .000 |
| Symptom Age | -0.209 | 0.104 | 4.087 | 1 | .043 | 0.811 |
| Arthritis (1) | -1.771 | 0.757 | 5.479 | 1 | .019 | 0.170 |
| Myalgia (1) | -0.990 | 0.532 | 3.460 | 1 | .063 | 0.372 |
| Chest Pain (1) | -1.642 | 0.826 | 3.953 | 1 | .047 | 0.194 |
| Fever | -18.574 | 15675.380 | 0.000 | 1 | .999 | .000 |
| Constant | 32.193 | 15675.381 | 0.000 | 1 | .998 | 95760419313587.530 |
